# Effects of Thresholding on Voxel-Wise Correspondence of Breath-Hold and Resting-State Maps of Cerebrovascular Reactivity

**DOI:** 10.3389/fnins.2021.654957

**Published:** 2021-08-24

**Authors:** Nooshin J. Fesharaki, Amy B. Mathew, Jedidiah R. Mathis, Wendy E. Huddleston, James L. Reuss, Jay J. Pillai, Edgar A. DeYoe

**Affiliations:** ^1^College of Health Sciences, University of Wisconsin-Milwaukee, Milwaukee, WI, United States; ^2^Department of Radiology, Medical College of Wisconsin, Milwaukee, WI, United States; ^3^Prism Clinical Imaging, Inc., Milwaukee, WI, United States; ^4^Neuroradiology Division, Russell H. Morgan Department of Radiology and Radiological Science, Johns Hopkins University School of Medicine, Baltimore, MD, United States; ^5^Department of Neurosurgery, Johns Hopkins University School of Medicine, Baltimore, MD, United States

**Keywords:** functional magnetic resonance imaging, neurovascular uncoupling, resting-state, amplitude of low-frequency fluctuations, breath-hold, cerebrovascular reactivity, brain mapping

## Abstract

Functional magnetic resonance imaging for presurgical brain mapping enables neurosurgeons to identify viable tissue near a site of operable pathology which might be at risk of surgery-induced damage. However, focal brain pathology (e.g., tumors) may selectively disrupt neurovascular coupling while leaving the underlying neurons functionally intact. Such neurovascular uncoupling can result in false negatives on brain activation maps thereby compromising their use for surgical planning. One way to detect potential neurovascular uncoupling is to map cerebrovascular reactivity using either an active breath-hold challenge or a passive resting-state scan. The equivalence of these two methods has yet to be fully established, especially at a voxel level of resolution. To quantitatively compare breath-hold and resting-state maps of cerebrovascular reactivity, we first identified threshold settings that optimized coverage of gray matter while minimizing false responses in white matter. When so optimized, the resting-state metric had moderately better gray matter coverage and specificity. We then assessed the spatial correspondence between the two metrics within cortical gray matter, again, across a wide range of thresholds. Optimal spatial correspondence was strongly dependent on threshold settings which if improperly set tended to produce statistically biased maps. When optimized, the two CVR maps did have moderately good correspondence with each other (mean accuracy of 73.6%). Our results show that while the breath-hold and resting-state maps may appear qualitatively similar they are not quantitatively identical at a voxel level of resolution.

## Introduction

The use of blood oxygenation level dependent (BOLD) functional MRI (fMRI) for presurgical brain mapping permits safer, more effective, surgery by allowing neurosurgeons to identify functional (eloquent) cortex close to a site of operable pathology (Hart, 2007; Pillai, 2010; Jenkinson et al., 2018; Vysotski et al., 2018). In this context, task-evoked fMRI activation serves as a biomarker for healthy brain tissue and can also indicate functional specificity (Ojemann, 1993; Hirsch et al., 2000; Petrella et al., 2006; Pillai, 2010; Genetti et al., 2013; Black et al., 2019). Consequently, a lack of fMRI response near or within a site of operable pathology is interpreted as a non-functional region that is safe for surgical removal (Hirsch et al., 2000; Orringer et al., 2012). Yet, this is not always correct. The neurovascular coupling mechanism that underlies task-evoked BOLD responses can be disrupted by focal brain pathology (e.g., tumor), causing reduced or absent fMRI signals despite the presence of functionally intact neurons (Holodny et al., 2000; Ulmer et al., 2004; Burke and Buhrle, 2006; Hou et al., 2006; Pak et al., 2017; Silva et al., 2017; Voss et al., 2019). Such “neurovascular uncoupling” (NVU) thereby causes false negatives on the fMRI task-activation maps (Ulmer et al., 2003; Pillai and Mikulis, 2014; Zaca et al., 2014; Para et al., 2017). The detection of NVU is critical for the safe, effective use of fMRI in presurgical planning (Pillai and Mikulis, 2014; DeYoe et al., 2015; Pak et al., 2017; Silva et al., 2017). If NVU is undetected, aggressive surgery could result in resection of eloquent cortex, thereby causing severe post-treatment neurological deficits and a diminished quality of life for the patient (Zaca et al., 2011; Rahman et al., 2016).

Unfortunately, it is not clearly understood how NVU occurs. Several pathophysiological factors have been proposed (Stanimirovic and Friedman, 2012; Pak et al., 2017). For example, NVU may occur in brain tumor patients, whereby an abnormal vascular response may be caused by tumor invasion of tissue surrounding cerebral blood vessels (Lee et al., 2009; Watkins et al., 2014). It has also been shown that an abnormally increased blood volume due to glioma-induced neovascularity may cause a ceiling effect on the BOLD signal (Hou et al., 2006). In contrast, a floor effect on the BOLD signal may result from an atypical amount of deoxygenated hemoglobin in the blood, perhaps due to a lack of sufficient blood flow (Fujiwara et al., 2004; Pillai and Mikulis, 2014). Indeed, a host of potential scenarios involving pathologic alterations in blood flow, volume, oxygenation and other factors could compromise the BOLD response despite preserved neural activity. (Here, for convenience, we use the term NVU to refer to the compromised BOLD response arising from any of these different factors.) Regardless of the exact causes of NVU, it is apparent that the offending pathology could logically occur at any point along the coupling cascade between neurons and the nearby microvasculature. However, there is currently no definitive evidence that NVU results from the disruption of the coupling cascade at early stages without also directly affecting the local vasculature (Watkins et al., 2014). Accordingly, one way to detect potential NVU is to directly test cerebrovascular reactivity (CVR) to changes in the blood concentration of carbon dioxide (CO_2_) (Pillai and Zaca, 2011; Zaca et al., 2011; Pillai and Mikulis, 2014; Zaca et al., 2014; Para et al., 2017). CVR can be mapped with fMRI by performing a task consisting of alternating periods of breath-hold and normal breathing (Kannurpatti et al., 2010; Murphy et al., 2011; Bright and Murphy, 2013; Tancredi and Hoge, 2013; Pillai and Mikulis, 2014; Peacock et al., 2016; Urback et al., 2017). Holding the breath is thought to slowly increase the concentration of blood CO_2_, which is known for its intrinsic vasodilatory effect (Chen and Anderson, 1997). The resultant rise in cerebral blood flow, ultimately, leads to a global BOLD response (Bandettini and Wong, 1997; Kastrup et al., 1999). Such a breath-hold challenge can be used as a direct test of vascular reactivity and can identify brain regions where such reactivity is defective (Pillai and Zaca, 2011; Zaca et al., 2011, 2014; Pillai and Mikulis, 2014; Iranmahboob et al., 2016; Voss et al., 2019). Typically, such a CVR test would be used in conjunction with task-fMRI to help verify that a zone of apparently unresponsive tissue is not actually harboring viable neurons. For example, if fMRI signals are absent for the hand representation of primary motor cortex, the lack of breath-hold induced CVR in this region would suggest that the task-fMRI response may have been compromised due to NVU rather than neural damage (Pillai and Zaca, 2011; Zaca et al., 2011). To verify true NVU (as opposed to dead brain tissue), one must demonstrate that the constituent neurons are still intact, which can be accomplished with an appropriate behavioral test (DeYoe and Raut, 2014; DeYoe et al., 2015).

Although the breath-hold approach for mapping CVR offers advantages in terms of the ease of implementation, reproducibility, and standardization (Bright and Murphy, 2013; Pillai and Mikulis, 2014; Liu et al., 2019), it does have some practical disadvantages. It is not suitable for patients who cannot reliably perform a breath-hold challenge, such as those who are unconscious, debilitated or too young to comply with the task demands. It is also unsuitable for patients with a large body habitus or chronic pulmonary disease (Laine et al., 1986; Kannurpatti et al., 2014). In practice, there can be significant variability in breath-hold performance across patients thereby limiting its utility unless combined with continuous monitoring of end-tidal CO_2_ (Thomason et al., 2005; Thomason and Glover, 2008; Bright and Murphy, 2013; Spano et al., 2013). Breath-hold following expiration can be more repeatable than breath-hold following inspiration. However, the former is not currently standard of practice because it is more challenging for patients (Thomason et al., 2007; Scouten and Schwarzbauer, 2008; Pillai and Mikulis, 2014; Wu et al., 2015). Finally, a separate breath-hold task requires additional MRI scan time and thus, higher expense for patients. Given these limitations, an approach based on a similar mechanism but without the need to perform any specific task may provide a better alternative (Chang and Glover, 2009; Jahanian et al., 2014; Kannurpatti et al., 2014; Golestani et al., 2016b).

A potential alternative to breath-hold for CVR mapping is to use data from resting-state fMRI (Wise et al., 2004; Birn et al., 2006, 2008; Biswal et al., 2007; Kannurpatti and Biswal, 2008; Kannurpatti et al., 2011, 2014; Golestani et al., 2015; Lipp et al., 2015; Tsvetanov et al., 2015, 2020; Golestani et al., 2016a,b; Jahanian et al., 2017; Liu et al., 2017; Chen and Gauthier, 2021; Pinto et al., 2021). In this approach, CVR maps can be derived from unique low-frequency (e.g., < 0.1 Hz) components of resting-state BOLD signals. Although resting-state fMRI is widely used for the mapping of multiple “functional networks” (Biswal et al., 1997a,b), similar signal components can also yield information about CVR (Wise et al., 2004; Golestani et al., 2016a, 2017; Liu et al., 2017; De Vis et al., 2018; Tong et al., 2019). Presumably, this is due to spontaneous fluctuations in blood CO_2_ during free breathing (Wise et al., 2004; Birn et al., 2006, 2008; Biswal et al., 2007; Liu et al., 2017; Tong et al., 2019). For example, CVR information can be obtained by computing the resting-state fMRI signal power within the 0.01–0.08 Hz frequency band, known as amplitudes of low frequency fluctuations (ALFF) (Yang et al., 2007; Zang et al., 2007). Research has also shown the suitability of investigating the time-delay of these fluctuations for assessing perfusion deficits (Amemiya et al., 2014; Khalil et al., 2017; Ni et al., 2017), and mapping altered brain connectivity (Jahanian et al., 2018). Recently, it has been shown that the total power of low-frequency resting-state BOLD signals (Yang et al., 2007; Zang et al., 2007) provides a metric that can detect tumor-induced NVU on presurgical brain maps (Agarwal et al., 2017, 2018).

Whether resting-state and breath-hold CVR maps are quantitatively equivalent has yet to be established—particularly, at the resolution of individual voxels and with a comprehensive treatment of threshold setting. Previous studies have shown that resting-state maps are quite similar to those obtained from breathing a controlled mixture of exogenous CO_2_ (Liu et al., 2017; De Vis et al., 2018). Moreover, both resting-state and breath-hold methods for scaling task-based BOLD signals, appear to be equivalent (Biswal et al., 2007; Kannurpatti and Biswal, 2008; Kannurpatti et al., 2011; Di et al., 2013; Kannurpatti et al., 2014). As part of a research study on the effect of low-frequency fluctuations on task-fMRI activation, Birn et al. (2006) demonstrated that BOLD amplitudes associated with free breathing at rest are similar to those induced by a breath-hold task. More recently, qualitative comparisons of breath-hold and resting-state patterns of CVR revealed overall good spatial correlation between the two maps (Lipp et al., 2015; Jahanian et al., 2017). Such evidence suggests that CVR metrics obtained from resting-state and breath-hold may arise from the same mechanism, which is thought to be CO_2_-induced variations in cerebral blood flow (Birn et al., 2006). If true, then the brain-wide CVR activation patterns of the two metrics should be nearly identical even at the resolution of individual voxels. Consequently, our primary goal was to quantitatively measure the accuracy of spatial correspondence of the breath-hold and resting-state CVR brain maps.

However, there is a significant complication in comparing breath-hold and resting-state CVR metrics in that their spatial patterns are critically dependent on the thresholding criteria used to identify valid responses. Despite being widely used in clinical (and academic) setting, thresholding remains an ongoing, practical issue for CVR (and many other) brain mapping techniques. Therefore, a second goal of this study was to comprehensively determine the effects of threshold settings on the accuracy of spatial overlap between the two CVR maps. In sum, we tested the prediction that breath-hold and resting-state metrics are quantitatively equivalent in spatial extent on a voxel-wise basis when threshold criteria are optimally matched. Establishing the optimum degree of spatial agreement between the two maps will help to clarify their relative potential for mapping vascular reactivity within the brain and will provide a quantitative basis for the development of imaging biomarkers for detecting NVU in clinical cases.

## Materials and Methods

### Participants

In this study, we recruited nine healthy adults (5 females, mean age 26.5 years, range: 23–35 years), with no history of neurological disease. A written, informed consent was obtained from each participant during an interview at the time of first enrollment in the study. Prior to data collection, all experimental procedures were approved by the Institutional Review Board of the Medical College of Wisconsin (protocol PRO00020109).

### Experimental Tasks

#### Breath-Hold

The breath-hold task was a block-design paradigm modeled after previous studies and adopted as standard of practice by the American Society of Functional Neuroradiology (Pillai and Mikulis, 2014; Black et al., 2017). Each epoch consisted of a 40 sec interval of normal respiration followed by a 4 s inhalation and then a 16 s breath-hold. This pattern was then repeated 4 times and a 20 s block of normal breathing was added at the end of the four cycles. A 4 s equilibration period was also added at the beginning of each run for a total scan time of 264 s (132 image volumes). In the MRI scanner, the three task phases were cued by visual text stimuli (“normal breathing,” “inhalation, “breath-hold”) to ensure consistent performance. The presentation of visual stimuli was via a video projector onto a back-projection screen viewed by the subject through a mirror system mounted on the head coil of the MRI system. To maximize each subject’s behavioral compliance, the breath-hold task was practiced in a training session prior to scanning. To help verify task performance, we also obtained real-time respiratory bellows data. Each participant performed the breath-hold task two times, and the average of the two was used for further analysis.

#### Resting-State

The resting-state fMRI images were collected during a 10 min interval for which subjects were instructed to remain relaxed and breathe normally with eyes open and fixed on a central marker on the screen. In addition, a 4 s equilibration period was added at the beginning of each run. Accordingly, a total of 302 image volumes (604 s) were obtained per run. The resting-state scan was repeated two times for each participant and was performed prior to the breath-hold experiment.

### Image Acquisition

MR imaging was performed with a 3 Tesla General Electric Signa Excite 750 MRI scanner at the Medical College of Wisconsin. For each subject, all images were acquired during a single session. T1-weighted anatomical images were acquired using a spoiled, gradient echo (SPGR) pulse sequence with the following parameters: TR = 8.2 ms, TE = 3.2 ms, 12° flip angle, field of view of 240 mm, matrix size of 256 × 224, and 180 axial slices with a 1 mm slice thickness. T2^∗^-weighted gradient echo, echo-planar imaging (EPI) fMRI scans were obtained using a 32-channel RF/gradient head coil with the following parameters: TR = 2,000 ms, TE = 30 ms, 77° flip angle, field of view of 240 mm, matrix size = 96 × 96, and a slice thickness of 5 mm. These parameters resulted in a raw voxel size of 2.5 × 2.5 × 5 mm, which was Fourier interpolated to 1.875 × 1.875 × 5 mm. A 4 s equilibration period was utilized for all functional MRI scans to allow for magnetization transients. We also assessed each subject’s alertness after each run by asking them to rate their alertness on a scale from 1 to 5, with 1 representing highly drowsy and 5 referring to full alertness. This provided an independent measure of potential data quality and was used as an additional exclusion criterion.

### Preprocessing

Data preprocessing was performed using AFNI^1^ (version 19.3.11, RRID:SCR_005927) (Cox, 1996). For each subject, the reconstructed datasets were preprocessed using a slightly modified version of AFNI’s afni.proc.py script as follows:

For each of the two breath-hold datasets, the equilibration period (4 s) plus the next 10 image volumes (20 s) were removed using AFNI’s 3dTcat. In addition, the last 20 volumes (40 s) of each breath-hold dataset were deleted due to a lack of sufficient time to entirely capture the last breath-hold response. This yielded a total of 100 images (200 s) for each of the two breath-hold datasets. For each of the two resting-state datasets, the equilibration period (4 s) was first removed and then the timeseries was truncated to 210 images (420 s, equivalent to a 7 min scan) and roughly matching the number of data points to be used to compute the breath-hold and resting-state metrics.

The next step was to bring all functional volumes of all runs into spatial alignment with each other and with the skull-stripped T1-weighted anatomical images. This was accomplished as follows: (a) AFNI’s 3dToutcount was used to identify a single time point whose associated image volume had a minimum number of voxels with extreme fMRI signal values (outliers). This volume was then used as the base for aligning all other image volumes in the timeseries after first removing any large signal spikes (AFNI’s 3dDespike). The alignment process was composed of two stages: the first being co-registration within each timeseries (AFNI’s 3dvolreg) and the second being registration to the anatomical dataset (AFNI’s align_epi_anat.py). In practice, these two alignments were accomplished in a single step by combining the two transformation matrices (AFNI’s 3dAllineate). The preceding alignment process generated six time-course signals corresponding to head movement along three directions of translation and three axes of rotation. The derivatives of the head motion signals were then also computed.

The final preprocessing step was to correct the functional datasets for the effects of head motion artifacts. For this step, the afni.proc.py script was set up so that: (1) Time points in the BOLD fMRI data having particularly aberrant values (Euclidean norm of the motion derivatives > 0.2), were identified. (2) Each voxel’s timeseries was then amplitude scaled to a range of 0–200 with a mean of 100 (AFNI’s 3dTstat and 3dcalc) (3) The head motion derivatives (see above) were used in a linear regression analysis using AFNI’s 3dDeconvolve to model head motion effects. (4) To obtain the final “cleaned” timeseries data, AFNI’s 3dTproject was used to first replace by interpolation the timepoints with excessive head motion identified in step 1, and then to regress out the regression matrix resulting from step 3. It is worth noting that we initially tried using both the original head motion signals and their temporal derivatives to correct datasets for motion. However, examination of the timeseries data revealed that this tended to exclude some legitimate breath-hold-related responses. This was not the case when using only the motion derivatives. Therefore, we used only the motion derivatives for head motion correction. The final cleaned and corrected time-course signals were then used for all subsequent analyses.

### Volumes of Interest

For each subject, two volumes of interest were created, one included both cerebral gray and white matter and the second included only cortical gray matter. Using AFNI and FSL^2^ (version: 6.0.2, RRID:SCR_002823), T1-weighted anatomical images were corrected for non-uniformity by removing possible shading artifacts (AFNI’s 3dUnifize). These were then used to generate a brain-only volume (AFNI’s 3dSkullStrip). The skull-stripped anatomical images were segmented into three different classes: gray matter, white matter, and cerebrospinal fluid (FSL’s FAST segmentation tool) (Zhang et al., 2001). A whole-brain volume consisting only of the gray and white matter classes was then further processed to eliminate all non-cortical structures. This was accomplished by creating a mask of to-be-excluded structures using Prism View^®^^3^ (RRID:SCR_016977) software in conjunction with the standard MNI ICBM152 template^4^ (RRID:SCR_005281) (Mazziotta et al., 1995). The mask covered cerebellum, brainstem, and several structures in the depths of the cerebrum, including corpus callosum, thalamus, hypothalamus, basal ganglia, hippocampus, and subthalamus. For each subject, the mask was then back transformed from MNI space to the subject’s native space. Finally, the mask was used to remove all the non-cortical structures from the whole-brain gray+white matter volume. We refer to the resulting volume as the gray/white matter volume of interest, “GWM-VOI.” In addition, the white matter volume was removed from the GWM-VOI so as to create a VOI consisting only of cortical gray matter, here termed the “GM-VOI.”

### Data Analysis

CVR data analysis was performed in three major steps: (Step 1) Compute metrics of CVR for each voxel in the GWM-VOI using (1a) the breath-hold data and (1b) the resting-state data. (Step 2) Optimize the spatial correspondence between CVR activation and cortical gray matter by testing a wide range of threshold settings. (Step 3) Optimize and compare the spatial overlap of the breath-hold and resting-state CVR patterns within cortical gray matter by testing a wide range of threshold combinations for both metrics.

#### Step 1a. Computing the Breath-Hold CVR Metric

An initial perusal of the data revealed significant individual differences in the latency and time-course of BOLD responses to the breath-hold task. Consequently, to ensure maximum sensitivity and accuracy for detecting each subject’s vascular response to the breath-hold task, we used a unique method to empirically measure the respiratory response waveform for each individual subject as follows: (i) an accurate estimate of the unique waveform of their respiratory response was first obtained; (ii) using this waveform, a breath-hold CVR metric was then computed for each voxel throughout the brain.

**FIGURE 1:**
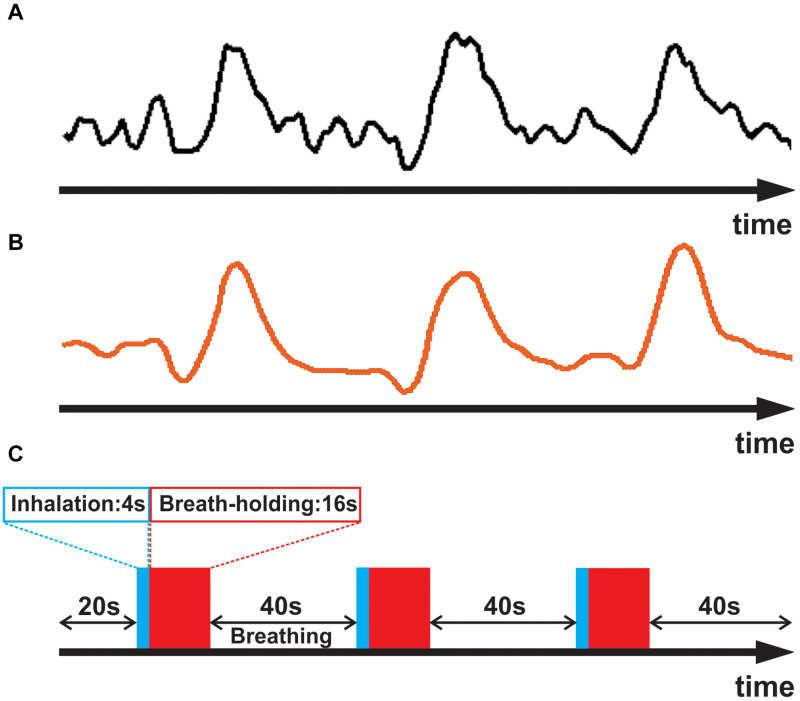
**(A)** Averaged empirical breath-hold BOLD time series for a representative subject. **(B)** The subject’s empirically estimated, personal respiratory response waveform. **(C)** Breath-hold task consisting of three epochs of 4 sec inhalation (blue), 16 s breath-hold (red), and 40 s normal breathing.

(i) To obtain an accurate estimate of the unique waveform of each subject’s breath-hold response, we began by identifying a sample of strongly responding voxels (an example waveform is shown in Figure 1A). This was accomplished computationally by cross correlating an initial estimate of the respiratory response with the empirical fMRI waveform for each voxel. The initial estimate was obtained by convolving the breath hold task timing (Figure 1C) with a generic respiratory response function (RRF), defined by Birn et al. (2008):

(1) $$R\,R\,F=0.6\,t^{2.1}\,e^{-t/1.5}-0.0023\,t^{3.54}\,e^{-t/4.25}$$

The top 1% most highly correlated empirical signals were then averaged, and the resulting waveform was smoothed with a three-point order statistic filter (Cox, 1996), defined as:

(2) $$Y_{t}=\left(0.7\right)\,m\,e\,d\,i\,a\,n\,\left(X_{t-1},X_{t},X_{t+1}\right)+\left(1.15\right)\,m\,a\,x\,\left(X_{t-1},X_{t},X_{t+1}\right)+\left(0.15\right)\,m\,i\,n\,\left(X_{t-1},X_{t},X_{t+1}\right)$$

In the above equation, for each time point (t) in the timeseries, the smoothing filter uses 3 input values of X_*t*–1_, X_*t*_, and X_*t*+1_ to compute one output value (Y_*t*_). Such filtering helped avoid having the fit be driven by spurious noise components, thereby yielding a better approximation of the subject’s respiratory response (Figure 1B). The smoothed timeseries was then used for the subsequent analyses.

(ii) To compute final breath-hold CVR metrics, each subject’s personalized respiratory response was used in a general linear regression analysis against the original empirical breath-hold time-series using AFNI’s 3dDeconvolve. This was performed by shifting the subject’s personalized respiratory response from 0 to 4 TR relative to each voxel’s empirical time-series and computing a regression fit coefficient (beta) for each time delay. Essentially, each beta coefficient was a measure of the amplitude of the fitted waveform. The time lag yielding the best fit of the subject’s respiratory response waveform to the timeseries was obtained for each voxel. Eventually, the beta coefficient corresponding to the voxel’s optimal time lag was then used as that voxel’s breath-hold CVR metric.

#### Step 1b. Computing the Resting-State CVR Metric

The resting-state fMRI signal power within a frequency band from 0.01 to 0.08 Hz, known as the Amplitude of Low Frequency Fluctuation (ALFF), was used directly as the resting-state metric for each voxel (Yang et al., 2007; Zang et al., 2007). We will refer to the ALFF metric as the resting-state “CVRe” metric, where the “e” implies “estimate.”

#### Step 2. Optimizing Overlap of CVR Maps With Gray Matter

The CVR activation pattern is thought to be confined predominantly to gray matter because of the latter’s high density of microvasculature (Thomas et al., 2014). Therefore, for each metric, we first searched for a threshold setting that would optimize the spatial correspondence between the CVR activation pattern and cortical gray matter, while also minimizing CVR activation appearing artifactually in white matter. This was done independently for both the breath-hold and resting-state maps by exploring a range of threshold criteria: Beta coefficient for breath-hold and ALFF for resting-state. At each threshold setting, voxels were classified as CVR responsive or not. Accordingly, for each threshold setting, voxels were classified as either: true positive (TP), true negative (TN), false positive (FP), or false negative (FN) as defined in Table 1A. Such a classification thus yielded one of the four binary combinations of 11, 00, 10, and 01 for each voxel. The analysis was restricted to the GWM-VOI and the resulting classification proportions were reported in a two-by-two contingency table (see Supplementary Figure 1A). At each threshold setting, the overlap between the resulting CVR activation pattern and cortical gray matter was assessed using accuracy (Acc) and Dice coefficients computed as:

(3) $$A\,C\,C=\frac{T\,P+T\,N}{T\,P+T\,N+F\,P+F\,N}$$

(4) $$D\,i\,c\,e=\frac{2\,T\,P}{2\,T\,P+F\,P+F\,N}$$

**TABLE 1:** Voxel-wise classification in **(A)**: CVR vs. gray matter comparisons, and **(B)** resting-state CVRe vs. breath-hold CVR comparisons.

(A) CVR vs. GM comparison			(B) Resting-state CVRe vs. Breath-hold CVR comparison		
	
Class	CVR	GM	Class	Resting-state CVRe	Breath-hold CVR
TP	1	1	TP	1	1
TN	0	0	TN	0	0
FP	1	0	FP	1	0
FN	0	1	FN	0	1

**GM, Gray Matter; TP, True Positive; TN, True Negative; FP, False Positive; FN, False Negative; 1 = Present; 0 = Absent.**

For each metric, this process was repeated over a full range of threshold settings to find the optimal threshold that maximized the accuracy of spatial overlap (as defined by Equation 3 above). A custom MATLAB program was used to perform this analysis.

#### Step 3. Comparing Breath-Hold CVR and Resting-State CVRe Maps

Having first established thresholds that optimized overlap of CVR maps with gray mater, we directly compared the breath-hold CVR and resting-state CVRe patterns with each other by again exploring a full range of potential thresholds for each metric: beta coefficient for breath-hold and ALFF for resting-state. To quantify the spatial overlap of the two maps, we again classified voxels according to the presence or absence of a response for each metric and for each different pair of threshold settings. Arbitrarily, we used the resting-state CVRe metric as a logical “predictor” of the breath-hold CVR metric. Voxels were classified as either: true positive (TP), true negative (TN), false positive (FP), or false negative (FN) as defined in Table 1B. The resulting voxel proportions for each threshold pair were reported as a two-by-two contingency table (see Supplementary Figure 1B) and used to compute correspondence accuracy [3] and Dice coefficient [4].

## Results

In accordance with the structure of our data analysis, we discuss the results in two main sections: (1) The association of CVR-responsive voxels with gray vs. white matter and (2) a voxel-wise comparison of the breath-hold CVR and resting-state CVRe maps with each other. In both instances, the comparisons were highly dependent on the threshold criteria used to identify CVR responsive voxels, but in both cases, clearly optimal threshold settings that maximized the spatial correspondence could be identified.

### CVR vs. Gray Matter

**FIGURE 2:**
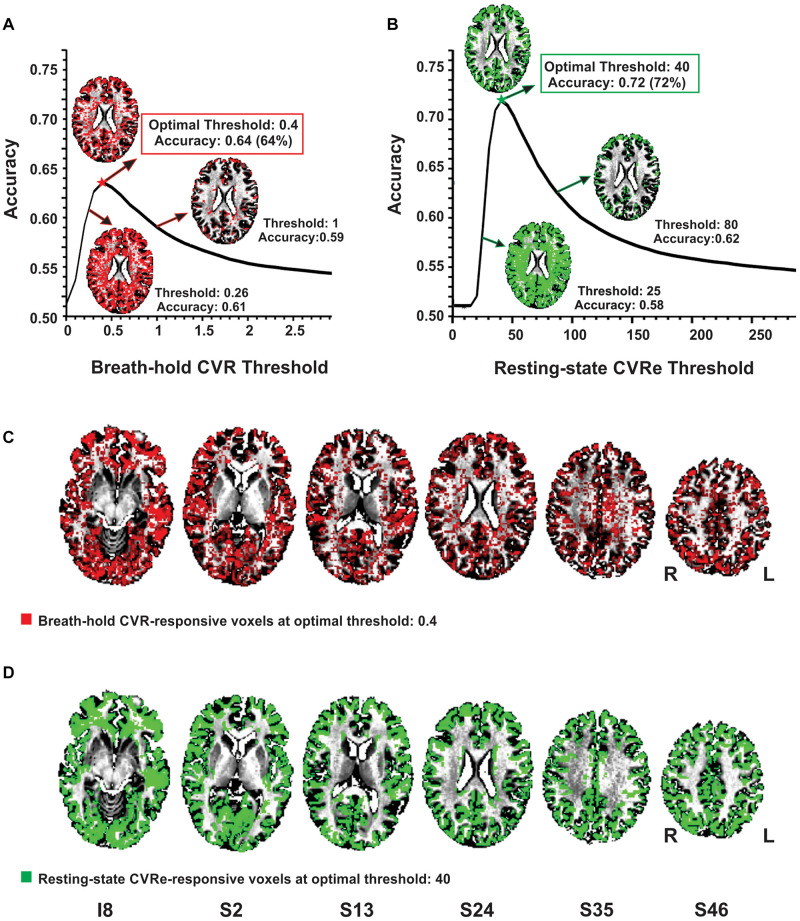
Optimizing thresholds to maximize accuracy of spatial overlap between CVR responses and gray matter. **(A)** Breath-hold CVR accuracy vs. threshold with example CVR brain patterns (red) for thresholds (beta coefficients) of 0.26, 0.4, and 1. **(B)** Resting-state CVRe accuracy vs. threshold with example CVRe brain patterns (green) for thresholds (ALFFs) of 25, 40, and 80. By decreasing the threshold from 1 to 0.26 in **(A)**, and from 80 to 25 in **(B)**, more complete coverage of the gray matter is obtained but at the cost of inappropriate labeling of white matter. **(C**,**D)** Axial brain maps for thresholds marked by stars in **(A,B)** that optimized overlap accuracy of gray matter with breath-hold CVR (**C:** red) and resting-state CVRe (**D:** green). Labels below images indicate relative slice position in mm (I, inferior; S, superior). Accuracy is defined in Equation 3. Slice right/left (R/L) orientation is radiologic standard.

As illustrated in Figures 2A,B, the accuracy of spatial correspondence between gray matter and both the breath-hold CVR and resting-state CVRe metrics varied dramatically with CVR threshold setting. The accuracy was calculated as the summation of CVR-responsive voxels in gray matter (true positive) and non-responsive voxels in white matter (true negative) relative to the total number of voxels (Equation 3 above). For both metrics, there was a clear optimum threshold that maximized the correspondence accuracy. The inset figures in Figures 2A,B make this relationship clearer by showing that at low thresholds there is inappropriate labeling of white matter, whereas at high thresholds, the labeling becomes too sparse in gray matter. At the threshold of maximum accuracy (Thresholds 0.4 and 40 in Figures 2A,B, respectively), labeling of gray matter is high yet inappropriate labeling of white matter is low. The resultant breath-hold CVR and resting-state CVRe maps at these optimum threshold settings (maximizing correspondence accuracy) are shown in Figure 2C (red voxels) and Figure 2D (green voxels), respectively.

Complete analyses for the CVR vs. gray matter comparisons (at optimal thresholds) are shown in Tables 2, 3 for the resting-state CVRe and breath-hold CVR metrics, respectively. For all subjects, paired *t*-tests revealed that CVR/gray-matter overlap accuracy and Dice scores were significantly higher for resting-state than for breath-hold (Acc: 71.7 ± 3.7 vs. 63.1 ± 4.7%, *p* < 0.01; and Dice: 74.2 ± 3.4 vs. 65.8 ± 4.3%, *p* < 0.01). Tables 2, 3 suggest that the better accuracy of resting-state reflects its relatively higher labeling within gray matter (TP: resting-state 40.8 ± 2.9%, vs. breath-hold 35.6 ± 3.3%, *p* < 0.01) and better avoidance of white matter (TN: resting-state 30.9 ± 3.3%, vs. breath-hold 27.6 ± 4.3%, *p* < 0.01). In other words, resting-state CVRe is more selectively localized to gray matter than breath-hold CVR. This is also qualitatively evident in Figure 2D vs. Figure 2C for a representative subject.

**TABLE 2:** Voxel-wise correspondence of resting-state CVRe and gray matter.

Voxel-wise correspondence of resting-state CVRe and gray matter						

Subject	TP (%)	TN (%)	FP (%)	FN (%)	Acc (%)	Dice (%)
#1	45	32	15	8	77	79.7
#2	41	35	17	7	76	77.4
#3	39	33	13	15	72	73.6
#4	37	30	22	11	67	69.2
#5	38	35	15	12	73	73.8
#6	42	32	18	8	74	76.4
#7	45	26	22	7	71	75.6
#8	41	27	21	11	68	71.9
#9	39	28	22	11	67	70.3
**Ave ± SD**	**40.8 ± 2.9**	**30.9 ± 3.3**	**18.3 ± 3.5**	**10 ± 2.3**	**71.7 ± 3.7**	**74.2 ± 3.4**

**Accuracy and Dice coefficients (right columns) were computed from the voxel classification data (middle columns) using Equations 3 and 4. Voxel classification procedures are described in section “Materials and Methods*.*” This analysis was limited only to the GWM-VOI. TP: True Positive; TN: True Negative; FP: False Positive; FN: False Negative; Acc: Accuracy; Dice: Dice Coefficient; Ave: Average; SD: Standard Deviation.**

**TABLE 3:** Voxel-wise correspondence of breath-hold CVR and gray matter.

Voxel-wise correspondence of breath-hold CVR and gray matter						

Subject	TP (%)	TN (%)	FP (%)	FN (%)	Acc (%)	Dice (%)
#1	36	31	17	16	67	68.6
#2	37	33	19	11	70	71.2
#3	36	28	19	17	64	66.7
#4	29	28	24	19	57	57.4
#5	34	30	20	16	64	65.4
#6	35	31	19	15	66	67.3
#7	41	24	24	11	65	70.1
#8	34	23	25	18	57	61.3
#9	38	20	30	12	58	64.4
**Ave ± SD**	**35.6 ± 3.3**	**27.6 ± 4.3**	**21.9 ± 4.1**	**15 ± 3**	**63.1 ± 4.7**	**65.8 ± 4.3**

**Accuracy and Dice coefficients (right columns) were computed from the voxel classification data (middle columns) using Equations 3 and 4. Voxel classification procedures are described in section “Materials and Methods*.*” This analysis was limited only to the GWM-VOI. TP: True Positive; TN: True Negative; FP: False Positive; FN: False Negative; Acc: Accuracy; Dice: Dice Coefficient; Ave: Average; SD: Standard Deviation.**

### Breath-Hold CVR vs. Resting-State CVRe

**FIGURE 3:**
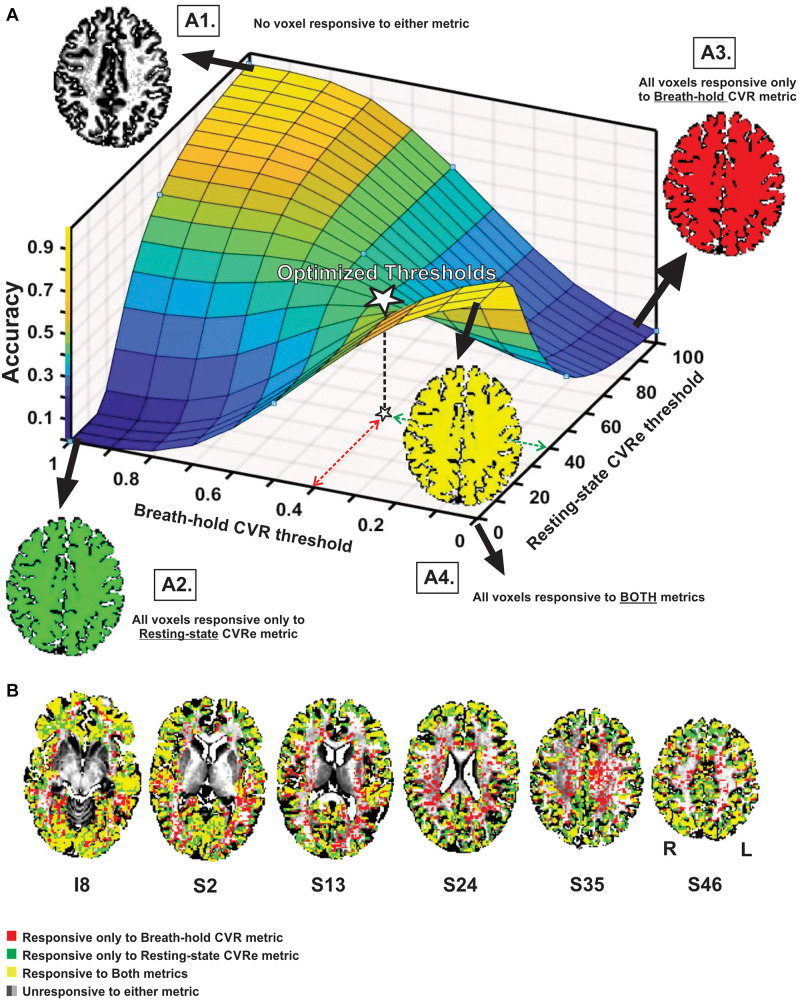
**(A)** Accuracy of spatial correspondence between breath-hold CVR and resting-state CVRe within cortical gray matter as a function of threshold (beta coefficient for breath-hold and ALFF for resting-state). Optimal threshold pair that maximized segregation of CVR responses to gray matter shown by dashed red and green arrows. Accuracy for those optimal thresholds is indicated by star. Moving threshold settings toward biased corners forces all voxels to be classified as CVR responsive to one (A2, A3), both (A4) or neither (A1) CVR metric. **(B)** Axial brain images with overlaid CVR activation maps at optimal threshold settings (0.4 and 40 for breath-hold and resting-state CVR maps, respectively). Labeling in gray and white matter is illustrated though accuracy measures pertain to the gray matter VOI only. Voxel color code indicated at lower left. Labels under slice images indicate relative slice position in mm (I, inferior; S, superior). Labels below the slice image in the right show the orientation of all slice images (R, right; L, left).

Figure 3 illustrates the effect of threshold setting on the accuracy of spatial correspondence between the breath-hold CVR and resting-state CVRe activation patterns. In this case, the accuracy calculations were restricted just to the cortical gray matter ROI (GM-VOI). (However, brain images in Figure 3 show labeling of both gray and white matter). The resulting 3-dimensional accuracy surface was saddle-shaped as illustrated in Figure 3A. The threshold settings that were determined in the previous analysis to optimize the correspondence of each CVR map with gray matter are indicated by the dashed arrows (red for breath-hold and green for resting-state) with the corresponding accuracy indicated by the star at mid-point of the saddle-shaped surface. The corners of the surface are associated with extreme threshold settings that force all voxels to be classified as active for both metrics (A4), only breath-hold CVR (A3), only resting-state CVRe (A2), or neither metric (A1). This was true for all subjects even though the precise shape of the surface varied somewhat from subject to subject.

As illustrated in Figure 3B, superimposing the two optimized patterns shows that they cover roughly the same regions but are not identical (matching voxels in yellow). The resting-state CVRe pattern (green + yellow) appears to demonstrate more complete coverage of gray matter with less encroachment into white matter. In contrast, the breath hold pattern (red + yellow) has excessive, inappropriate labeling of white matter.

Table 4 shows the complete analysis quantifying the spatial correspondence between the breath-hold CVR and resting-state CVRe activation patterns for all subjects. In this case the computations were restricted only to voxels within gray matter (GM-VOI). At the level of individual voxels, the mean accuracy of spatial correspondence between the two metrics was 73.6 ± 3.4%, ranging from 69 to 81%. Similarly, the Dice coefficients ranged from 77 to 88.3% with an average of 82.2 ± 3%.

**TABLE 4:** Voxel-wise correspondence of resting-state CVRe and breath-hold CVR.

Voxel-wise correspondence of resting-state and breath-hold CVR patterns						

Subject	TP (%)	TN (%)	FP (%)	FN (%)	Acc (%)	Dice (%)
#1	65	9	20	6	74	83.3
#2	72	9	13	6	81	88.3
#3	59	14	17	10	73	81.4
#4	52	17	25	6	69	77
#5	56	18	18	8	74	81.2
#6	62	11	23	4	73	82.1
#7	65	9	22	4	74	83.3
#8	62	13	19	6	75	83.2
#9	60	10	18	12	70	80
**Ave ± SD**	**61.4 ± 5.7**	**12.2 ± 3.5**	**19.4 ± 3.6**	**6.8 ± 2.4**	**73.6 ± 3.4**	**82.2 ± 3**

**Resting-state CVRe was used as a predictor of Breath-hold CVR. Accuracy and Dice coefficient metrics of spatial correspondence (right columns) were computed from the voxel classification data (middle columns) using Equations 3 and 4. Voxel classification procedures are described in section “Materials and Methods*.*” This analysis was limited only to the gray matter ROI (GM-VOI). TP: True Positive; TN: True Negative; FP: False Positive; FN: False Negative; Acc: Accuracy; Dice: Dice Coefficient; Ave: Average; SD: Standard Deviation.**

### Unthresholded Breath-Hold CVR vs. Resting-State CVRe

**FIGURE 4:**
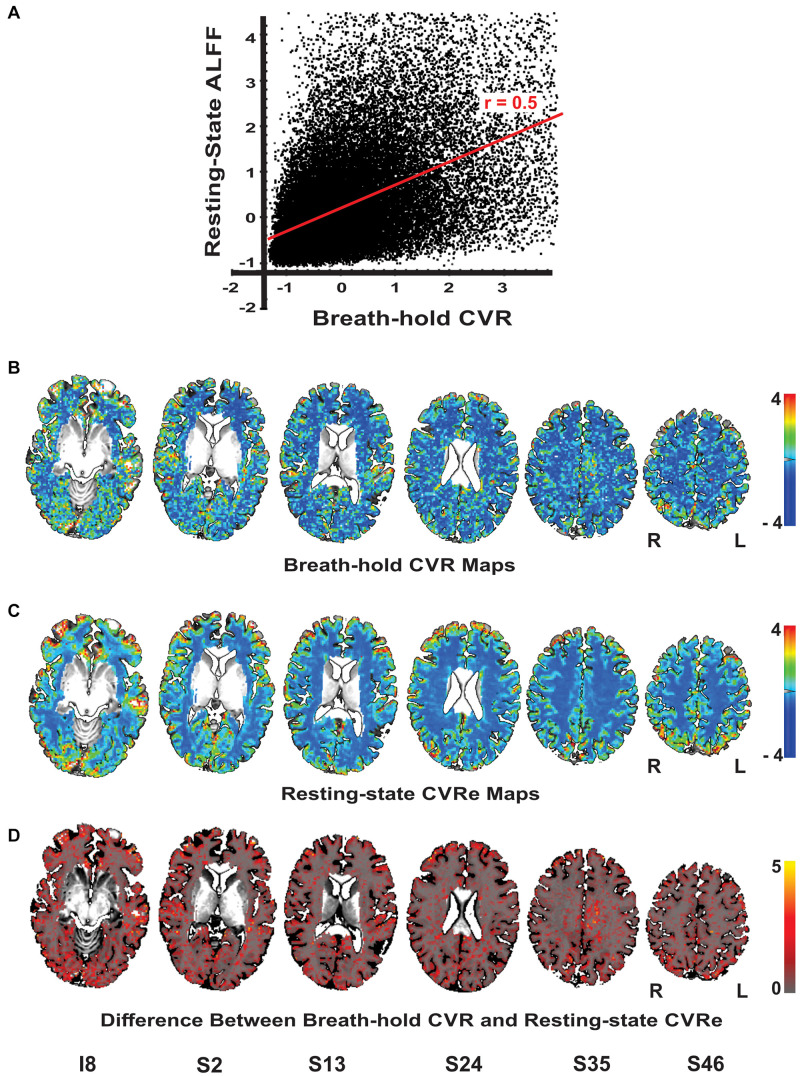
**(A)** Scatterplot of the unthreshold resting-state and breath-hold CVR metrics for a representative subject. Each point represents one voxel in the cortical gray matter. Full range of data has been clipped to 95% of voxels. The two metrics are correlated with a correlation coefficient of 0.50. **(B–D)** Axial brain images with overlaid unthresholded maps of breath-hold CVR **(B)**, resting-state CVRe **(C)**, and difference metric **(D)**. Voxel color code indicated at right. Labels under slice images indicate relative slice position in mm (I, inferior; S, superior). Slice right/left (R/L) orientation is radiologic standard.

Although our primary goal in this study was to compare breath-hold and resting-state metrics as they have typically been utilized in the clinical field with thresholding, it is informative to briefly compare the unthresholded metrics. Figure 4A illustrates a scatter plot of the unthresholded metrics (after normalization of their respective amplitude ranges) for a representative subject. There was a moderate linear correlation of the two metrics (*r* = 0.50) but with significant scatter. This result was consistently obtained for all subjects (mean correlation = 0.53). The spatial distribution of the unthresholded metrics within the GWM-VOI are illustrated for a representative subject in Figures 4B,C. To contrast the two distributions, we computed the difference between the two metrics for each voxel (after normalization of their respective amplitude ranges). Figure 4D illustrates the brain pattern of this difference metric. Consistent with our original analysis, this shows that there are no obvious large regions of significant mismatch between the breath-hold CVR and resting-state CVRe metrics. The local voxel-wise differences are more or less randomly distributed throughout gray matter.

## Discussion

The primary goal of this study was to compare a resting-state metric to a widely accepted breath-hold CVR metric to determine if their activation patterns were spatially equivalent at a voxel level of resolution appropriate for presurgical brain mapping. Since local blood flow regulation within cerebral cortex is largely controlled by gray matter microvasculature, we first identified threshold settings for each metric that optimized gray matter coverage while minimizing artifactual spread into white matter. Having spatially optimized CVR responses in this manner, we then quantified the degree of spatial overlap of the two metrics on a voxel-by-voxel basis.

The main findings of this study are: (1) The correspondence between breath-hold CVR and resting-state CVRe maps and their association with cortical gray matter are highly dependent on the CVR threshold settings. (2) Both breath-hold and resting-state metrics have clearly optimal thresholds that maximize their coverage and segregation relative to gray vs. white matter. (3) At optimal threshold settings, the spatial pattern of activation for the two metrics differs significantly at a voxel level of resolution with the resting-state metric providing better coverage and segregation to cortical gray matter. (4) At the voxel level, the two metrics had moderate overlap accuracy, ranging from 69 to 81% overlap with a mean of 73.6%. Despite this modest correspondence at the voxel scale, the patterns were nevertheless qualitatively consistent on a more global scale as reported previously by others (Lipp et al., 2015; Jahanian et al., 2017). These results allow us to reject our prediction that “the breath-hold and resting-state metrics are quantitatively equivalent in spatial extent on a voxel-wise basis when threshold criteria are optimally matched.”

Our results showed that, when optimized, cross-metric correspondence accuracy was relatively modest ranging from 69 to 81% across our nine subjects. One might ask why this result seems somewhat at odds with previous reports of good correspondence using breath-hold and/or resting-state metrics as indicators of healthy vs. impaired CVR (Pillai and Zaca, 2011; Zaca et al., 2011, 2014; Iranmahboob et al., 2016; van Niftrik et al., 2016; Agarwal et al., 2017; Jahanian et al., 2017; Liu et al., 2017; De Vis et al., 2018). The critical effect of optimizing threshold selection in the present study is likely the most important difference between our approach and those of previous studies, where CVR thresholds were often set by z-score significance as in task-fMRI or to obtain full coverage of gray matter (Lipp et al., 2015; Jahanian et al., 2017). Our approach of optimizing threshold settings for each individual was motivated by clinical utility in which a patient-specific approach is paramount. This is somewhat different from previous studies that were focused on comparing metrics across subjects (Lipp et al., 2015).

We felt that it was important to examine the correspondence of our CVR metrics at a voxel resolution of 2.5 × 2.5 × 5 mm which is generally consistent with current clinical studies. In contrast previous studies have often used larger voxel sizes (Golestani et al., 2016b; Jahanian et al., 2017) and/or coarse spatial smoothing with a 4–8 mm FWHM kernel (Pillai and Zaca, 2011; Zaca et al., 2011, 2014; Lipp et al., 2015; Iranmahboob et al., 2016; van Niftrik et al., 2016; Agarwal et al., 2017; Liu et al., 2017; De Vis et al., 2018). This tends to produce artifactually uniform and continuous maps thereby obscuring their true discontinuous nature as documented in the present study. Heavy smoothing also will obscure smaller CVR anomalies making the detection of large (relatively rare) dropouts more reliable at the expense of spatial precision. The potentially smallest detectable CVR dropout is unclear, in part, because the precision of microvascular control is poorly defined (Attwell et al., 2010), but may reflect the 1–2 mm spacing of individual arterioles that penetrate cortical gray matter (Duvernoy et al., 1981; El-Bouri and Payne, 2016). If so, routine use of smaller imaging voxels and less smoothing might enhance the detection of more subtle CVR defects that may prove to be of clinical relevance.

It should be stressed that our study was focused on providing a quantitative measure of how well the patterns of the two CVR metrics agreed for individual subjects (ultimately patients), not on proving that the spatial patterns or the level of agreement were the same across individuals. Consequently, the most relevant statistical issue was the rate of agreement across the thousands of voxels within each individual. While such a descriptive statistic is sufficient for this purpose, we also did test multiple subjects. This was to illustrate the consistency of correspondence across a sample of individuals, but not to test whether it was identical across subjects. Nevertheless, we did find that the rate was surprisingly consistent, varying by only 3.4% across our sample of 9 subjects (Table 4).

A second important goal of our study was to critically examine the effects of threshold settings on the spatial properties and correspondence of breath-hold and resting-state metrics. This was motivated by the fact that thresholding of CVR and fMRI maps is ubiquitous in the literature and in current clinical practice. Yet, its effects are rarely explored systematically and comprehensively. In the present study, we explored wide ranges of threshold settings in order to show that the spatial correspondence of the two metrics varies significantly, but in understandable ways, with changes in threshold. We feel that this is an important caveat to the interpretation of previous studies that examined the spatial patterns of the two metrics. Typically, an arbitrary or poorly justified threshold was used for the classification of voxels as being CVR responsive or not (Pillai and Zaca, 2011; Zaca et al., 2011, 2014; Lipp et al., 2015; Golestani et al., 2016b; Jahanian et al., 2017; Liu et al., 2017; De Vis et al., 2018; Cohen and Wang, 2019). Indeed, it was not uncommon for the threshold settings in previous studies to be adjusted to obtain full coverage of gray matter while ignoring potentially inappropriate coverage of white matter. It was also quite common for CVR maps to be thresholded in the same manner as task-based fMRI activation despite having different physiological bases (Agarwal et al., 2017; Jahanian et al., 2017). Such approaches can result in an inflated rate of false positive CVR responses, thereby underestimating the occurrence of zones having compromised CVR (Lipp et al., 2015; Liu et al., 2017), which in a surgical scenario, can increase the risk of ablation of viable brain tissue. Notably, even after optimizing threshold settings, the accuracy of overlap of our CVR metrics with gray matter tended to be modest (~70–80%) at its maximum. This reflected a combination of incomplete labeling of gray matter with some inappropriate CVR activation within white matter. However, this is precisely the goal of using an unbiased criterion to optimize the threshold selection. Note that simply maximizing sensitivity does not explicitly take into account the concurrent effect on proliferation of false positives. Indeed, arbitrarily lowering the CVR threshold to obtain more complete labeling of gray matter (i.e., to maximize detection of true positives) will unavoidably increase inappropriate labeling of white matter (i.e., increased rate of false positives) resulting in a reduction of overall correspondence accuracy (Figures 2A,B). To be clear, however, we do not advocate that thresholding of clinical CVR maps is necessarily the best way to analyze the data. Rather, our purpose here is to ensure that if practitioners do elect to threshold their brain maps, that they are aware that arbitrary selection of the threshold settings can produced a biased map.

Interestingly, our exploration of a wide range of threshold settings for the cross-metric CVR comparison produced a saddle-shaped accuracy surface, wherein the optimal thresholds obtained in the CVR vs. gray matter comparison were located at, or very close to, the center of the surface (white star in Figure 3A). From a methodological standpoint, the saddle area represents the best accuracy attainable without artificially pushing the thresholds toward the biased corners of the surface where extreme settings artificially force all voxels to be classified as responsive or not (Figures 3A1–4). For example, inset Figure 3, A4 illustrates how threshold values at the front corner force all voxels to be true positive (TP), meaning that they are all classified as responsive for both resting-state CVRe and breath-hold CVR metrics thereby forcing the correspondence accuracy to be 1.0 (100%). In other words, the more that threshold settings are forced toward the corners, the more biased the accuracy value becomes. We stress that this is an important point that both clinicians and researchers should note: Arbitrary adjustment of thresholds is very likely to create a biased CVR map!

Besides the importance of thresholding in creating unbiased CVR maps, we also found that the optimal thresholds for best segregation of CVR responses to gray matter corresponded precisely with the thresholds that were independently optimized for unbiased comparison of the two metrics with each other. From a conceptual standpoint, a likely explanation is that maximizing the CVR spatial overlap relative to gray vs. white matter may also effectively optimize the patterns to reflect the spatial distribution of the underlying vascular structures that produce CVR signals and are known to be concentrated in gray matter. Note that while the fortuitous match between the two sets of thresholds does not prove our assumption that true CVR signals should primarily arise from the highly vascularized gray matter, it is consistent with its general validity.

Although we comprehensively explored the effects of thresholding, we also cross-checked our overall conclusions about the spatial characteristics of the two metrics by examining unthresholded CVR maps and an unthresholded difference metric (Figure 4). We showed that the breath-hold and resting-state metrics are indeed quantitatively correlated though somewhat loosely (Figure 4A). Thus, both the unthresholded maps and analysis are consistent with our conclusion that, overall, the two metrics are grossly similar in amplitude and spatial distribution but can vary significantly at the voxel level.

A potentially important limitation of this study is that it focuses on breath-hold CVR and resting-state CVRe metrics that are both thought to primarily test the vascular responsiveness to variations in blood CO_2_ levels. As a potential indicator of disrupted CVR, vasoactivation represents only one stage in the complex cascade of physiological events linking neuronal activity to an fMRI response. In principle, neurovascular coupling could fail due to pathological effects on earlier stages of the cascade prior to the vascular smooth muscle response. Whether this can actually occur in clinical practice is unknown but physicians who are responsible for interpreting the results of CVR testing should be made aware of this potential caveat.

Our prediction that breath-hold and resting-state CVR metrics would produce matching brain maps was based on the premise that both arise from ongoing changes in blood CO_2_ levels. Although, our observations of significant cross-metric correspondence are consistent with this scenario, the lack of a closer correspondence at the voxel level suggests that additional neuronal and/or physiological factors (such as cardiac pulsation or respiration) may cause some variability in the two maps (Tsvetanov et al., 2015; Golestani et al., 2016a, 2017; Chu et al., 2018; Tsvetanov et al., 2020). For example, artifactual labeling of white matter in the breath-hold CVR maps may partly reflect head motion that typically accompanies the active breath-hold task thereby reducing overlap accuracy. (This may also account for the more selective and complete coverage of gray matter by the resting-state metric.) On the other hand, resting-state ALFF may not exclusively reflect CVR alone, but also may contain contributions from other neuronal and/or physiological factors such as cardiac pulsation and respiration (Makedonov et al., 2013; Tsvetanov et al., 2015; Golestani et al., 2016a, 2017; Agarwal et al., 2017; De Vis et al., 2018). However, if such factors influence both metrics in the same way then this may potentially enhance the correspondence between the two though not necessarily due to CVR (Tsvetanov et al., 2015, 2020). In particular, we were concerned that the correspondence between our two metrics might be affected by tissue-specific physiological factors such as whole-brain, global signals, cerebrospinal fluid pulsation, etc. However, we found that explicit removal of tissue-based signal components did not alter our results or conclusions. Importantly, the inclusion of additional (neuronal and/or physiological) signal components in a CVR metric does not necessarily invalidate its use as an indicator of compromised hemodynamics. Indeed, for the purpose of assessing fMRI activation integrity in the clinical context of surgical guidance, a qualitative rather than fully quantitative measure of CVR can be sufficient (Pillai and Mikulis, 2014).

We were also concerned that both breath-hold and resting-state patterns reflect a significant amount of random noise which will necessarily reduce the maximum obtainable spatial correspondence. To quantitatively assess this latter noise factor, we estimated the maximum attainable concordance by comparing two independent samples of breath-hold CVR data with each other. Similarly, we also compared two independent samples of resting-state CVRe data. For a sample of 5 of our original subjects, the resulting mean correspondence accuracies were 64% for breath-hold CVR and 80% for resting-state CVRe while the mean cross-metric correspondence accuracy was 71.4% (see Supplementary Table 1, 2). The lower self-concordance of the breath-hold metric may reflect patient variation in task performance (Bright and Murphy, 2013) and the artifactual effects of head motion which typically accompany an active breath-hold task. This may also be the primary factor limiting accuracy in the cross-metric correspondence.

Another consideration was that the lower self-correspondence of the breath-hold data might reflect a lack of sufficient sensitivity to detect all of the valid responses. In principle, this could occur if the breath-hold respiratory response is temporally unique for each person. Indeed, there is evidence that breath-holding induces BOLD changes that are relatively slower than for free breathing at rest (Birn et al., 2008). To circumvent this concern, we separately measured the respiratory response waveform for each individual subject and used it as a template for detecting each subject’s breath-hold CVR signals throughout the brain. We even tested each subject’s personalized respiratory response waveform with a range of delays to ensure maximum sensitivity for each voxel.

Although the primary focus of the present study was to compare two popular breath-hold CVR and resting-state CVRe metrics having clinical potential, we stress that they do not necessarily represent the best or most accurate measures of CVR that have been, or could be, devised. Indeed, these two metrics, as used in common practice, provide a qualitative rather than precisely quantitative test of cerebrovascular reactivity. Yet, for clinical use as a marker of potential fMRI “dropout” in presurgical brain maps, this can be sufficient. Fortunately, the path to a more quantitative and potentially informative metric is being actively pursued. Passive arterial CO_2_ and/or respiratory-volume signals can provide a more quantitative estimate of CVR from resting-state data (Birn et al., 2006; Golestani et al., 2016b; Jahanian et al., 2017). A number of recent studies have also explored latency maps computed from resting-state data in healthy controls and patients with cerebrovascular disorders (Amemiya et al., 2014; Christen et al., 2015; Aso et al., 2017; Khalil et al., 2017; Ni et al., 2017; Tong et al., 2017). Such latency maps are thought to reflect both the arrival of cerebral blood flow and the reaction time of vasculature to changes in blood CO_2_ (De Vis et al., 2018), thus providing alternate metrics of blood perfusion and vascular reactivity.

From the standpoint of clinical utility, it is important to note that the significantly better, more specific, coverage of gray matter by the resting-state CVRe metric may offer some modest advantage over the breath-hold technique as a biomarker for testing fMRI activation integrity. The use of resting-state has the advantage of not requiring a separate breath-hold task. Moreover, as the resting-state fMRI technique becomes more widely accepted as an alternative to conventional task-based fMRI for brain mapping, both functional and CVR maps can be derived from the same resting-state dataset. Since an important long-term goal is to use these metrics clinically for detecting potential NVU in patients facing invasive brain surgery, the results of this study provide a strong base for more extensive clinical tests with patients having a variety of operable brain pathologies. Such clinical studies will be needed to verify that both breath-hold CVR and resting-state CVRe metrics are able to reliably detect NVU in patients with ongoing brain pathology.

## Conclusion

Our quantitative comparison suggests that breath-hold CVR and resting-state ALFF maps are spatially similar, but not identical at a voxel level of resolution. Their mutual correspondence as well as their accurate association with cerebral gray matter are critically dependent on the threshold settings chosen to identify valid CVR-responsive voxels and this must be considered when interpreting CVR brain maps. The resting-state pattern appears to segregate more accurately relative to gray vs. white matter and has more complete coverage of gray matter than the breath-hold pattern. Additionally, the higher correspondence between two independent samples of the resting-state maps compared to two samples of the breath-hold maps suggests that the resting-state CVRe metric may be a modestly more reliable biomarker while also avoiding the need for consistent task performance with potentially compromised clinical patients. It should be noted that both metrics provide incomplete and variable labeling of gray matter at the resolution of individual voxels. Previous reports of reliable detection of CVR loss in clinical patients typically have used subjective threshold settings and high levels of spatial smoothing that obscure this variability, resulting in an unknown loss of sensitivity for detecting small, yet potentially significant CVR anomalies.

## Data Availability Statement

The post-processed data that support the findings of this article will be available on request from the corresponding author. The raw MRI data won’t be publicly available due to ethical restrictions related to subject identifiability.

## Ethics Statement

The studies involving human participants were reviewed and approved by the Institutional Review Board of the Medical College of Wisconsin (protocol PRO00020109). The patients/participants provided their written informed consent to participate in this study.

## Author Contributions

NF, AM, and JM carried out the experiment and performed the computations. NF and ED verified the analytical methods and contributed to the interpretation of the results. NF wrote the manuscript with support from AM and ED. ED, JR, and JP conceived the original idea. ED supervised the project. All authors provided critical feedback and helped shape the manuscript.

## Conflict of Interest

JR was CEO of Prism Clinical Imaging, Inc. ED was a board member and part owner of Prism Clinical Imaging, Inc. The remaining authors declare that the research was conducted in the absence of any commercial or financial relationships that could be construed as a potential conflict of interest.

## Publisher’s Note

All claims expressed in this article are solely those of the authors and do not necessarily represent those of their affiliated organizations, or those of the publisher, the editors and the reviewers. Any product that may be evaluated in this article, or claim that may be made by its manufacturer, is not guaranteed or endorsed by the publisher.

## Footnotes

1
http://afni.nimh.nih.gov/afni

2
https://fsl.fmrib.ox.ac.uk

3
http://www.prismclinical.com

4
http://en.wikibooks.org/wiki/MINC/Atlases
