# Supplementary material for: Effects of Thresholding on Voxel-Wise Correspondence of Breath-Hold and Resting-State Maps of Cerebrovascular Reactivity
Source: Front Neurosci. 2021 Aug 24;15:654957. doi: 10.3389/fnins.2021.654957 (PMC8421787; doi:10.3389/fnins.2021.654957)
Supplement: Supplementary file 1 [file Data_Sheet_1.docx]

**SupplementaL Materials**

**Two by Two Contingency Tables**


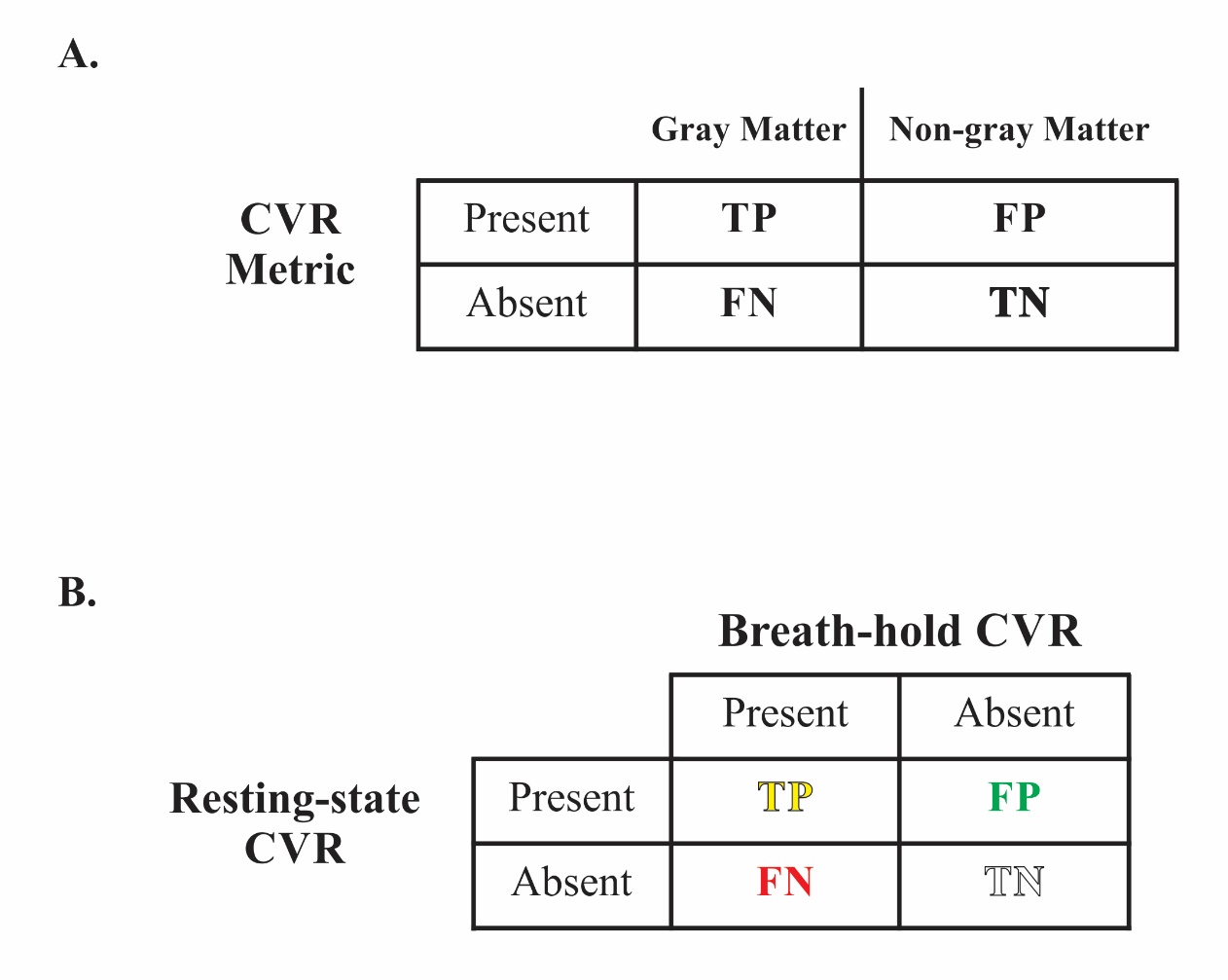


**Figure 1S.** **Two by Two Contingency Tables** for **A:** breath-hold or resting-state CVR vs gray matter comparisons, and **B:** breath-hold vs resting-state CVR comparisons. TP: true positive, TN: true negative, FP: false positive, FN: false negative. Colors in B correspond to voxel colors in Figure 3B.

**Cerebrovascular Reactivity Metric Self-Comparison Control**

For 5 subjects, we compared two independent samples of breath-hold cerebrovascular reactivity (CVR) data to each other and did the same for two samples of resting-state CVR data. These data were subjected to the same predictive value analysis described in the main text. Supplemental Tables 1S and 2S provide an abbreviated listing of just the Accuracy and Dice coefficients for comparison with the results from the cross-metric analysis. For our sample of 5 subjects, the mean cross-metric correspondence accuracy was 71.4%.

**Table 1S.** Voxel-Wise Correspondence of Two Independent Breath-hold CVR Patterns. Accuracy and Dice coefficient were computed using equations #3 and # 4.

**Voxel-Wise Correspondence of Two Independent Breath-hold CVR Patterns**

| **Subject** | **Acc** | **Dice** |
| --- | --- | --- |
| **#1** | 65 | 66 |
| **#2** | 63 | 64 |
| **#3** | 61 | 61 |
| **#4** | 66 | 68 |
| **#5** | 63 | 64 |
| **Avg ± SD** | **63.6 ± 1.9** | **64.6 ± 2.6** |

**Table 2S.** Voxel-Wise Correspondence of Two Independent Breath-hold CVR Patterns. Accuracy and Dice coefficient were computed using equations #3 and # 4.

**Voxel-Wise Correspondence of Two Independent Resting-state CVR Patterns**

| **Subject** | **Acc** | **Dice** |
| --- | --- | --- |
| **#1** | 89 | 90 |
| **#2** | 77 | 79 |
| **#3** | 80 | 86 |
| **#4** | 80 | 85 |
| **#5** | 76 | 76 |
| **Avg ± SD** | **80.4 ± 5.1** | **83.2 ± 5.6** |

**Schematic of Methodology**


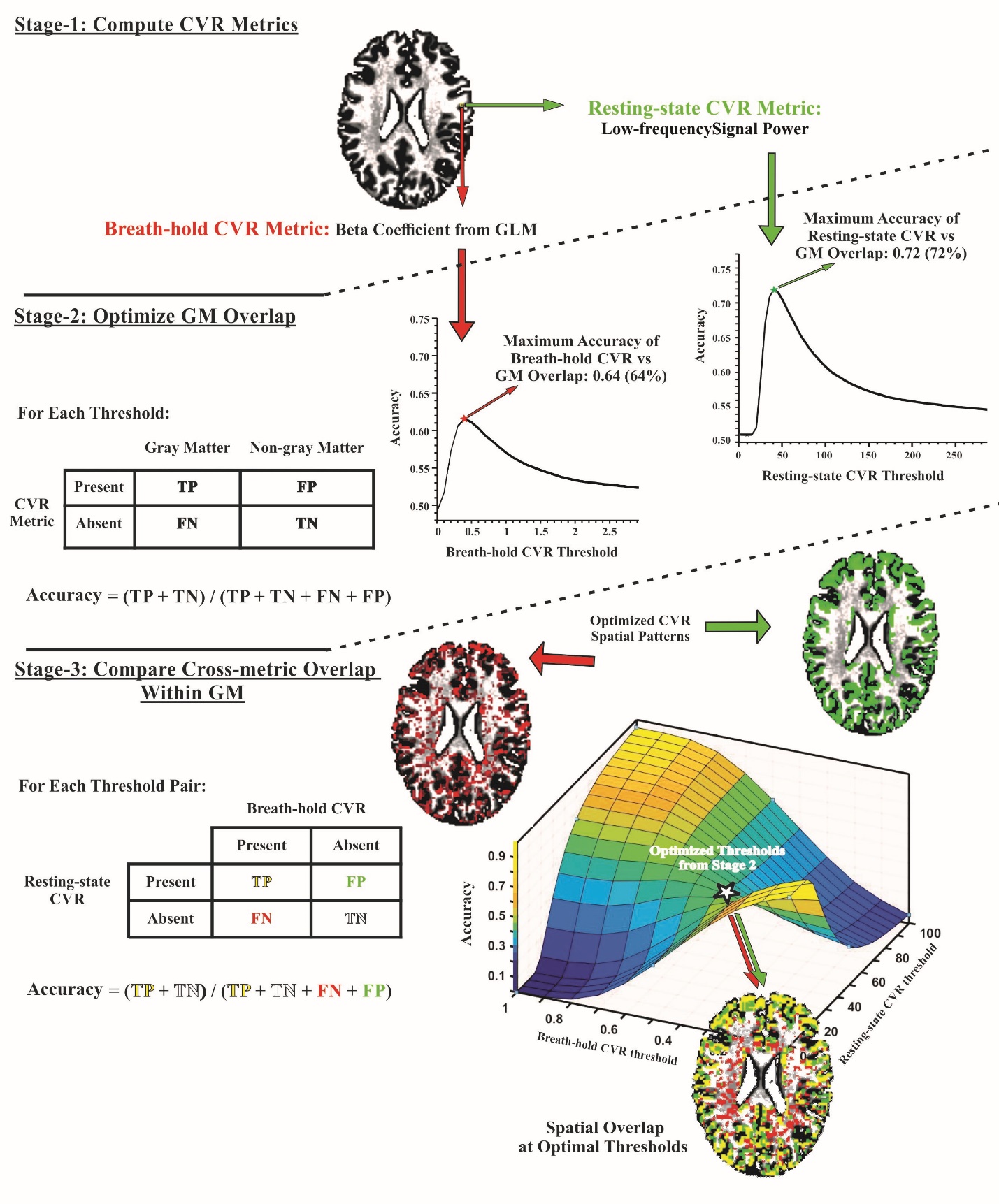


**Figure 2S.** **Schematic of Methodology.** CVR: cerebrovascular reactivity; GM: gray matter; GLM: general linear model; TP: true positive; TN: true negative; FP: false positive; FN: false negative. Voxels responsive to the resting-state, breath-hold, and both metrics are shown in green, red, and yellow, respectively.

**RSFA vs. ALFF**

**
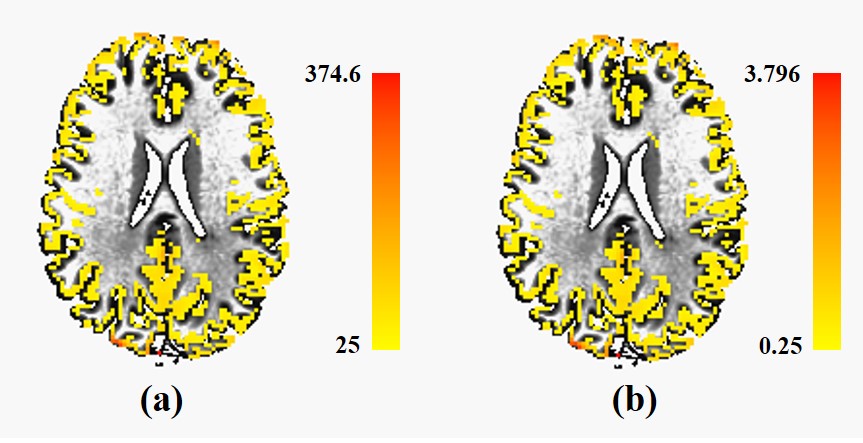
**

**Figure 3S**. Brain labelling patterns of **(a)** ALFF vs. **(b)** RSFA metrics. Thresholds were selected to maximize accuracy of spatial overlap with gray matter while minimizing inappropriate labeling of white matter. The two patterns are virtually indistinguishable (but not identical) and overlap accuracies were comparable (overlap accuracy: ALFF 72% vs. RSFA 73%).
